# A Novel Presentation of Refractory Immune Thrombocytopenia in Anti-synthetase Syndrome: A Case Report

**DOI:** 10.7759/cureus.42199

**Published:** 2023-07-20

**Authors:** Robert D Nguyen, Hao H Tong, Siobán Keel

**Affiliations:** 1 Medicine, University of Washington School of Medicine, Seattle, USA; 2 Internal Medicine, University of Washington School of Medicine, Seattle, USA; 3 Hematology, University of Washington School of Medicine, Seattle, USA

**Keywords:** autoimmune, interstitial lung disease, immune thrombocytopenia, anti-synthetase syndrome, thrombocytopenia

## Abstract

Anti-synthetase syndrome (AS) is a rare autoimmune disorder classified among the idiopathic inflammatory myopathies and is characterized by antibodies directed against aminoacyl-transfer RNA synthetases and the presence of myositis, interstitial lung disease, ±arthritis. Here, we report, for the first time, immune thrombocytopenia (ITP) in a patient with AS. This case reports a new association of AS with ITP and highlights the utility of identifying the underlying driver in secondary ITP to guide therapy.

## Introduction

Anti-synthetase syndrome (AS) is an autoimmune disorder characterized by autoantibodies against one of the many aminoacyl-transfer RNA synthetases, most commonly anti-Jo-1 antibodies and least commonly anti-PL7 antibodies [1]. In the literature, AS characterized by anti-PL7 antibodies (the target antigen is threonyl-tRNA synthetase) commonly presents with interstitial lung disease (ILD) (77%), myositis (75%), and arthritis (56%) [1,2]. Other clinical features of AS include fever, pericarditis, Raynaud’s phenomenon, and mechanic’s hands. Compared to other idiopathic inflammatory myopathies like dermatomyositis and polymyositis, AS has a higher prevalence of ILD, and among anti-PL7-positive patients and in particular those with co-existing anti-Ro52 antibodies, a higher frequency of rapidly progressive ILD is observed compared to AS patients lacking these autoantibodies [1,2].

## Case presentation

**Figure 1:**
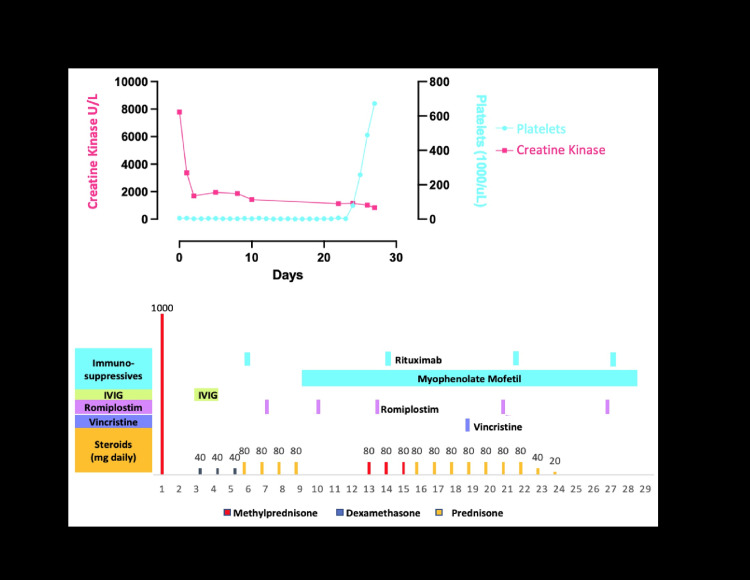
Patient treatment timeline Timeline of treatment and platelet counts and creatine kinase values. D1 indicates initial presentation to an outside hospital. Rituximab dosing: 375 mg/m^2^ IV weekly; MMF dosing:  500 mg PO BID on days 9-17, increased to 500 mg/1000 mg on day 18, 1000 mg BID on day 20, and 1500 mg BID on day 24; IVIG dosing:  1 g/kg IV on days 3 and 4; romiplostim dosing: 2 mcg/kg on days 7 and 10, 7 mcg/kg on days 14 and 27, and 10 mcg/kg on day 21; vincristine dosing: 1.5 mg/kg on day 19.

A 42-year-old male presented to an outside hospital with epistaxis and petechiae in the context of a six-month history of myalgias and arthralgias. He was found to have a creatine kinase level of 1,684 U/L (normal ref: 200-395 U/L) and a platelet count of 1 × 10^9^/L (150-450 × 10^9^/L), with normal coagulation studies (Figure 1). He was started on high-dose steroids, IVIG, and rituximab for a presumed diagnosis of ITP without platelet count response and transferred to our tertiary care center for refractory ITP management (Figure 1).

**Figure 2:**
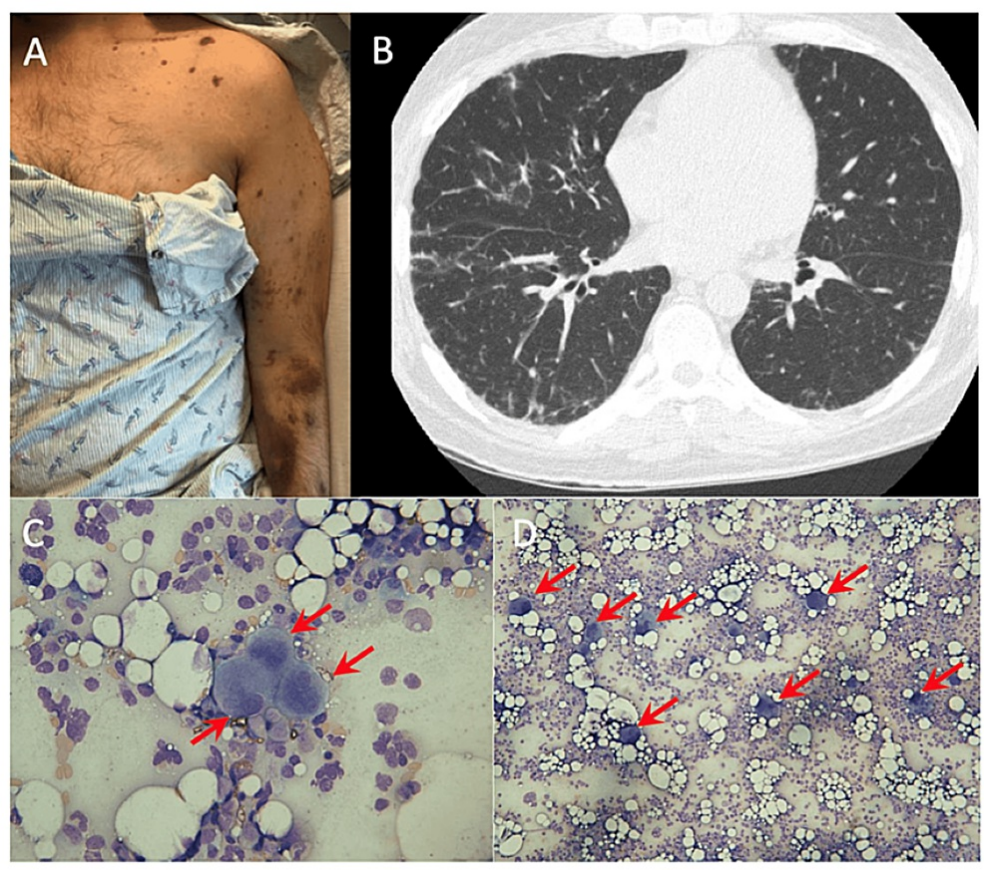
Patient skin exam, imaging, and peripheral blood smear (A) Photograph of the patient’s left arm on initial presentation demonstrating multiple ecchymoses. (B) High-resolution lung CT scan demonstrating fibrotic non-specific interstitial pneumonia that can be seen in the setting of myositis-related ILD. (C) and (D) Bone marrow aspirate smear (600× and 430× magnification, respectively) demonstrating adequate megakaryocytes (arrows). CT, computed tomography; ILD, interstitial lung disease.

On arrival, his vitals were normal and physical examination was notable for wet purpura, nasal packing, and scattered ecchymoses and petechiae (Figure 2A). Labs revealed a platelet count of 1 × 10^9^/L, white blood cell count of 17.22 × 10^9^/L (5-10 × 10^9^/L), and hemoglobin of 11% (13-18%). Autoimmune labs were notable for a positive antineutrophil antibody test (ANA) with a cytoplasmic speckled pattern and positive anti-SSA, anti-PL7, and anti-Ro52 antibodies (Table 1). Findings on computer tomography (CT) of the chest were consistent with fibrotic non-specific interstitial pneumonia, concerning myositis-associated ILD (Figure 2B). CT of the abdomen/pelvis was unremarkable and findings on an MRI of the right thigh were consistent with myositis. A peripheral blood smear showed severe thrombocytopenia with normal platelet size without clumping. Bone marrow aspirate and biopsy showed a normocellular marrow for age with trilineage hematopoiesis, megakaryocytes with a spectrum of maturation (Figure 2C, 2D), and minor immunophenotypic changes in the myeloid lineage by flow cytometry. The patient was refractory to platelet transfusions. In the setting of arthralgias, myositis, ILD, positive PL7 antibody, and refractory thrombocytopenia, the patient was diagnosed with AS and secondary ITP.

**Table 1:** Autoimmune and infectious panel ANA, antinuclear antibodies; CMV, cytomegalovirus; EBV, Epstein-Barr virus.

Autoantibody/antibody	Result
Immune panel	
ANA	Positive, cytoplasmic speckled pattern
Anti-SSA antibody	Positive
Anti-CCP antibody	Negative
Antiphospholipid antibody	Negative
Aldolase	Negative
C3	Normal
C4	Normal
Myositis-specific auto-antibodies	
Anti-EJ antibody	Negative
Anti-Jo1 antibody	Negative
Anti-Ku antibody	Negative
Anti-MDA5 antibody	Negative
Anti-Mi-2 antibody	Negative
Anti-NXP2 antibody	Negative
Anti-OJ antibody	Negative
Anti-PL7 antibody	Positive
Anti-PL-12 antibody	Negative
Myositis-associated auto-antibodies	
Anti-Ku antibody	Negative
Anti-Ro52 antibody	Positive
Infectious panel	
COVID-19 PCR	Negative
Adenovirus antibody	Negative
Coccidioides IgG and IgM antibodies	Negative
CMV IgM antibody	Negative
EBV antibody	Negative
Hepatitis B antibody	Negative
Hepatitis C antibody	Negative
Histoplasma urine antigen	Negative
HIV antibody	Negative
*Helicobacter pylori* stool antigen	Negative
Parvovirus B19 PCR	Negative
Tuberculosis QuantiFERON	Negative
Varicella zoster PCR	Negative

Hospital course****

Given his diagnosis of AS with ILD and myositis, the patient was continued on steroids. Additionally, he was started on mycophenolate mofetil (MMF) and continued on rituximab therapy, and both were thought to be optimal for ILD in the context of AS [3,4]. MMF was also thought to be the appropriate therapy for his refractory secondary ITP, given his lack of response to steroids or IVIG [4] in combination with high-dose thrombopoietin (TPO) receptor agonist therapy [5,6]. Provided his evidence of hemostatic compromise, he was additionally maintained on platelet transfusions and antifibrinolytic therapy until he demonstrated evidence of a platelet count response (Figure 1). He received a single dose of vincristine therapy during his period of severe thrombocytopenia and clinical evidence of hemostatic compromise given this agent’s rapid onset of action in ITP [7]. Unfortunately, he did not respond to vincristine. His platelet counts ultimately improved from 3 (day 24) to 79 (day 25), likely in response to high-dose TPO receptor agonist therapy.

## Discussion

ITP is well described in patients with lupus, dermatomyositis, and polymyositis [1]. However, this is the first reported case of ITP in a patient with AS. A case of thrombotic thrombocytopenic purpura in a patient with AS has been previously reported and was attributed to the proinflammatory state triggering the generation of autoantibodies against ADAMTS13 [8]. In ITP secondary to lupus, IgG autoantibodies targeting platelet glycoproteins GPIIb/IIIa, TPO or its receptor (MPL) have all been described [3]. We suspect that an analogous pathophysiology led to ITP in our AS patient. The observation that our patient initially presented with progressive musculoskeletal symptoms and shortness of breath, at least four months before developing refractory epistaxis and mucosal bleeding from thrombocytopenia, suggests that his AS preceded his ITP.

Fortunately, there is significant overlap in the therapies used to treat AS and ITP (steroids, IVIG, MMF, rituximab). This case highlights the importance of a multidisciplinary approach between rheumatology and hematology in therapy selection for secondary ITP due to an underlying autoimmune disorder. Our patient’s ITP was refractory to standard initial ITP therapies, IVIG and steroids [9,10]. The patient required steroids, MMF, and rituximab for his AS with myositis and ILD. MMF and rituximab are prioritized as upfront treatments in patients with inflammatory myopathies with ILD [4,5]. While rituximab has activity in newly diagnosed ITP, the durability of response is low and its utility in the upfront setting should be carefully weighed against its risks [11]. Rituximab was not continued in this patient for an ITP indication, but rather for his AS. This patient was initially referred to our center for splenectomy consideration for refractory ITP. In the current era, splenectomy is very rarely (if ever) recommended in ITP and should be reserved for extremely refractory ITP which has not responded to multiple single agents or combination therapy. Moreover, given this patient’s need for immunosuppressive therapy for his AS, we were particularly reluctant to consider splenectomy for ITP. Based on the timing of platelet recovery and his ongoing response to therapy (two weeks after his hospital discharge, his platelet count rose to 1,250 × 10^9^/L), we suspect that his ITP responded to high-dose TPO receptor agonist therapy. TPO receptor agonist therapy was recommended to avoid additional immunosuppression in our patient [12].

## Conclusions

ITP may be a rare manifestation of AS. Future studies into the shared pathophysiology between ITP and AS may inform optimal treatment approaches. Combination drug therapy with anti-CD20 monoclonal antibodies and TPO receptor agonists seems to be effective in mitigating platelet autoimmune peripheral destruction and bone marrow platelet proliferation in ITP secondary to AS.
